# Cable bacteria at oxygen‐releasing roots of aquatic plants: a widespread and diverse plant–microbe association

**DOI:** 10.1111/nph.17415

**Published:** 2021-05-21

**Authors:** Vincent V. Scholz, Belinda C. Martin, Raïssa Meyer, Andreas Schramm, Matthew W. Fraser, Lars Peter Nielsen, Gary A. Kendrick, Nils Risgaard‐Petersen, Laurine D. W. Burdorf, Ian P. G. Marshall

**Affiliations:** ^1^ Section for Microbiology Department of Biology Center for Electromicrobiology Aarhus University Ny Munkegade 116 Aarhus C DK‐8000 Denmark; ^2^ School of Biological Sciences The University of Western Australia 35 Stirling Highway Crawley WA 6009 Australia; ^3^ The UWA Oceans Institute The University of Western Australia 35 Stirling Highway Crawley WA 6009 Australia; ^4^ Ooid Scientific White Gum Valley WA 6162 Australia; ^5^ Max Planck Institute for Marine Microbiology Celsiusstraße 1 Bremen D‐28359 Germany

**Keywords:** aquatic plants, cable bacteria, plant–microbe interaction, rhizosphere, rice, roots, seagrass, sulfide

## Abstract

- Cable bacteria are sulfide‐oxidising, filamentous bacteria that reduce toxic sulfide levels, suppress methane emissions and drive nutrient and carbon cycling in sediments. Recently, cable bacteria have been found associated with roots of aquatic plants and rice (*Oryza sativa*). However, the extent to which cable bacteria are associated with aquatic plants in nature remains unexplored.
- Using newly generated and public 16S rRNA gene sequence datasets combined with fluorescence *in situ* hybridisation, we investigated the distribution of cable bacteria around the roots of aquatic plants, encompassing seagrass (including seagrass seedlings), rice, freshwater and saltmarsh plants.
- Diverse cable bacteria were found associated with roots of 16 out of 28 plant species and at 36 out of 55 investigated sites, across four continents. Plant‐associated cable bacteria were confirmed across a variety of ecosystems, including marine coastal environments, estuaries, freshwater streams, isolated pristine lakes and intensive agricultural systems. This pattern indicates that this plant–microbe relationship is globally widespread and neither obligate nor species specific.
- The occurrence of cable bacteria in plant rhizospheres may be of general importance to vegetation vitality, primary productivity, coastal restoration practices and greenhouse gas balance of rice fields and wetlands.

## Abstract 2

See also the Commentary on this article by Cardini & Malkin, **232**: 1897–1900.

## Introduction

Plant root–microorganism interactions are crucial for plant health and increasing crop yields (Berendsen *et al*., 2012; Pérez‐Montaño *et al*., 2014). The root microbiome is composed of microorganisms that inhabit the endosphere (inside of the roots), the rhizoplane (surface of the roots) and the rhizosphere, the soil around the root that is influenced by root processes (Gaiero *et al*., 2013). In most freshwater and coastal sediments, as well as in rice fields, oxygen only diffuses into the uppermost millimetres (Revsbech *et al*., 1980; Nicolaisen *et al*., 2004; Sobek *et al*., 2009). Below the oxic zone, sulfate is reduced to sulfide, which can accumulate to phytotoxic levels (Lamers *et al*., 2013). Radial oxygen loss (ROL), which is the release of oxygen from plant roots, is facilitated by root‐internal lacunar tissue called the aerenchyma. ROL has been hypothesised to be a defence mechanism against toxic sulfide through abiotic and biotic oxidation (Brodersen *et al*., 2015).

Filamentous, sulfide‐oxidising bacteria, called cable bacteria, uniquely influence aquatic geochemistry as they enable the spatially separated reactions of sulfide oxidation and oxygen reduction through a flow of electrons along their internal wires over centimetre distances (Pfeffer *et al*., 2012; Meysman *et al*., 2019). This process is called electrogenic sulfide oxidation and is usually associated with the uppermost 1–3 cm of sediment. Oxygen from the water column has been recognised as one of the dominant controlling factors for successful cable bacteria growth (Burdorf *et al*., 2018; Marzocchi *et al*., 2018; Liu *et al*., 2021). Known cable bacteria belong to the class Deltaproteobacteria (now redefined as phylum Desulfobacterota (Parks *et al*., 2018) and are divided into two described candidate genera: *Candidatus* Electrothrix found in marine systems and *Candidatus* Electronema found in freshwater systems (Trojan *et al*., 2016). Cable bacteria absolute abundances can be determined using fluorescence *in situ* hybridisation (FISH), while relative abundances can be derived from 16S rRNA gene sequencing of the microbial community (Trojan *et al*., 2016; Li *et al*., 2020; Liu *et al*., 2021). The latter however can result in unreliable estimates as extraction of DNA from cable bacteria can be difficult due to the rigid filament structure (Trojan *et al*., 2016), and biases can be introduced during the analysis by technical limitations and possible variations in gene copy number among cells (Bálint *et al*., 2016; Zinger *et al*., 2019).

Recently, cable bacteria have been found in mangrove sediments (Burdorf *et al*., 2016) and around oxygen‐releasing roots of seagrass (Martin *et al*., 2019, 2018a), saltmarsh *Salicornia europaea*, freshwater plants (including *Littorella uniflora* and *Lobelia cardinalis*) and rice (Scholz *et al*., 2019). It has been proposed that this may protect the roots against sulfide intrusion (Martin *et al*., 2018a) and indirectly suppress methane emissions (Scholz *et al*., 2020). The studies that investigate cable bacteria around roots of aquatic plants have so far been predominantly based on laboratory grown plants (Martin *et al*., 2018a; Scholz *et al*., 2020, 2019) and the natural occurrence of plant‐associated cable bacteria is unknown.

Given that oxygen from roots and sulfide from sulfate reduction are common traits in aquatic sediments and waterlogged soils, we hypothesised that the plant–cable bacteria relationship occurs globally and involves diverse plant species and life stages in marine, freshwater and terrestrial environments such as lakes and rice fields. To explore this hypothesis:

1. We used 16S rRNA gene amplicon sequencing of newly generated and public datasets to search for the occurrence of cable bacteria in 14 seagrass species from four continents, five freshwater plants from lakes in Europe, eight salt marsh plants in China and rice from USA, China, India and Vietnam.
2. We then visualised and quantified cable bacteria around plant roots collected from the field using FISH.
3. We also used FISH in combination with oxygen planar optodes to determine whether cable bacteria inhabit rhizospheres of seagrass seedlings and whether they co‐occur with oxygen release in these developing rhizospheres.

## Materials and Methods

### Description of field sites

Samples for 16S rRNA gene sequencing and FISH analysis were taken from roots of aquatic plants and sediments at various locations. Samples from Lake Cadagno, Switzerland, were collected in September 2018. Samples of *Potamogeton* sp. and *Equisetum* sp. were taken from the littoral zone (46°32′54.0″N, 8°42′49.2″E) and samples of *Isoetis* sp. were sampled from shallow water ways of the wetland with photosynthetic biofilms in immediate vicinity to Lake Cadagno (46°32′52.9″N, 8°42′03.1″E). Samples of *Littorella* *uniflora* were taken at Lake Hampen (July 2019; 56°01′11.2″N, 9°22′40.7″E) and Lake Knud (July and October 2019; 56°06′03.9″N, 9°44′45.6″E), Denmark. In addition, samples of *Pilularia globulifera* were taken from Lake Knud, Denmark (July 2019; 56°06′19.6″N, 9°45′35.2″E). Samples of the two seagrass species, *Zostera marina* and *Zostera noltii*, were collected in Aggersund, Denmark (March 2019; 56°59′52.3″N, 9°17′51.0″E) and Arcachon Bay, France (May 2019; 44°42′55.1″N 1°07′50.1″W), respectively (Supporting Information Tables S1, S2).

In the meromictic (water layers do not intermix) Lake Cadagno, constant inflow of sulfate‐rich groundwater causes high sulfide concentrations in the porewater, up to 900 µM (Putschew *et al*., 1996; Xiong *et al*., 2019). In the oligotrophic Lake Hampen (Christensen & Sørensen, 1986) no sulfide was evident, judged from white sandy sediment without any odour. In Lake Knud, the presence of sulfide was recognised by a foul smell and dark‐coloured sediment in certain spots caused by the presence of iron sulfides. The sites in Aggersund and Arcachon were both located in marine environments.

### Sample collection at field sites

Intact sediment cores with plants were retrieved with a spade or plastic cores (inner diameter > 10 cm), and either transported back to the laboratory or directly subsampled in the field. The sediment cores were opened and roots with adhering sediment or the sediment from the oxic/anoxic transition zone around oxygen‐releasing roots were sampled. Samples for nonplant associated cable bacteria were collected from the oxic/anoxic transition zone at the surface of the bulk sediment (at least 3 cm away from the plant shoots).

Oxygen‐releasing roots were identified by red iron oxide precipitates on the root surface, depletion of black iron sulfide around the roots, or by oxygen‐sensitive planar optode measurements. Samples taken in the field for DNA extraction were transported on ice or dry ice and stored at −80°C. Samples for FISH were either mixed with ethanol (v/v, 50 : 50) and stored at −20°C in the laboratory until further processing within a few weeks, or fixed in a 4% formaldehyde solution in phosphate‐buffered saline (PBS) for 2 h. Fixed roots were then washed twice in 1× PBS and stored in 1× PBS/96% ethanol (v/v, 30 : 70) at −20°C until further processing. The presence of sulfide was qualitatively assessed from the colour of the sediment and the odour.

### Collection of seagrass seeds and aquaria design

**Fig. 1:**
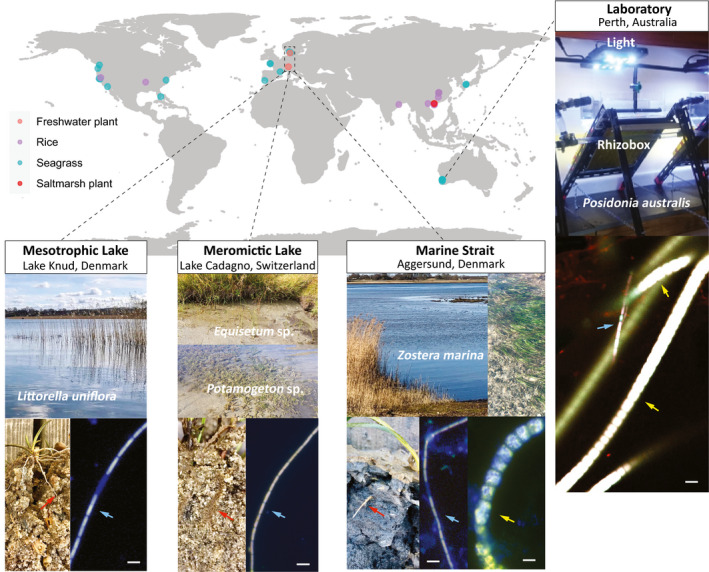
Global distribution of samples with confirmed plant‐associated cable bacteria (based on 16S rRNA gene sequences) and fluorescence *in situ* hybridisation (FISH) detection of cable bacteria. Samples from 16S rRNA gene sequences include the rhizosphere, rhizoplane, endosphere or paddy soil. Red arrows in photographs point towards the roots. Blue and yellow arrows in images from FISH point towards thin and thick cable bacteria filaments, respectively. Lower FISH images show hybridisations with probes DSB706, specific for Desulfobulbaceae (red) and EUB‐MIX targeting most bacteria (green), counterstained with DAPI (blue). Cable bacteria cells appear whitish/yellow from overlay of the two probes and DAPI stain. The upper right FISH image shows hybridisations with the probes Delta 495a‐c specific for Deltaproteobacteria (green), EUB‐MIX (red) and DSB706 (blue). Cable bacteria appear whitish from overlay of all three probes. Bars in FISH images, 5 µm. The photograph of the laboratory incubation shows the rhizoboxes that were used for monitoring root growth and oxygen release from *P*. *australis* seedlings (Supporting Information Fig. S1b).

Fruits of the seagrass *Posidonia australis* were collected directly from adult plants at a maximum depth of 5 m from Woodman Point, Western Australia in November 2016 (32°08′17.7″S, 115°45′48.2″E). Sediment samples were collected from the same site using plastic cores (inner diameter: 10 cm). Fruits were transported back to the laboratory and placed in an aerated aquarium (*c*. 350 l, *S* = 34). Fruits were kept under natural light with constant flow and aeration until the fruits naturally dehisced (*c*. 1 wk; Fig. S1a). Healthy germinated seeds were then selected and planted into three specially designed measuring aquaria containing sieved (2 mm) sediment from the sample site. The sediment was a silty sand with a porosity of 46.8 ± 4.5% v/v (measured by saturation), bulk density of 1.4 ± 0.1 g cm^−3^ and organic matter content of 2.6 ± 0.05% dry weight (combustion for 4 h at 450°C; Erftemeijer & Koch, 2001). The measuring aquaria, here designated rhizoboxes, were designed to monitor root growth and oxygen dynamics in the rhizosphere of *P*. *australis* seedlings and consisted of 10‐mm thick acrylic with a larger upper compartment for shoot growth (300 mm W × 200 mm H × 150 mm D), and a thinner lower compartment (300 mm W × 330 mm H × 20 mm D) containing the sediment (Fig. 1 (upper right panel), S1b). The lower compartment was angled at 45° and was equipped with a detachable front plate fitted with a thin (*c*. 0.5 cm) strip of neoprene that was held together with screws to ensure it was watertight. The front plate was fitted with an oxygen planar optode (see section below) and the entire lower compartment was covered in insulating foil to prevent interference from ambient light. The aquaria operated as a recirculating system when temperature and salinity were maintained at 24°C and *S* = 34 in a larger separate reservoir containing filtered seawater. Plants were grown under an irradiance of 250 mol E m^−2^ s^−1^ under a 14 h : 10 h, light : dark cycle for 4 wk.

### Rhizobox imaging and sampling of seagrass seedlings

The two‐dimensional oxygen distribution in the rhizosphere of *P*. *australis* seedlings was mapped using oxygen‐sensitive planar oxygen optodes as described in Martin *et al*. (2018a). The oxygen planar optodes were sufficiently transparent to discern roots growing behind them, which allowed the continual monitoring of root growth and length of oxic area (mm) along the roots. After 3 wk of incubation, the rhizoboxes were imaged each day for 1 wk. The extent of the oxygenated area around the roots of *P*. *australis* seedlings was quantified at day 28 of the incubation and expressed as maximum oxygen concentration of the oxic area, as well as total width of ROL and oxic length of roots of the same age from three replicate plants (each in a separate rhizobox) using Imagej software (Rueden *et al*., 2017). Total width of ROL was taken from the widest point and included the diffusion of oxygen from both sides of the root.

At day 28 of incubation, three replicate roots (one plant per rhizobox) were collected for FISH analysis. For FISH sampling, rhizoboxes were carefully opened and single roots from a seedling from each separate rhizobox were cut at the root tip (*c*. 5 mm, which includes the root cap and elongation zone with no root hairs) and at the root hair zone (*c*. 20–40 mm from the root tip). The unwashed root segments were immediately placed into a 4% formaldehyde (v/v) solution in PBS and fixed overnight at 4°C. Roots were then washed in 1× PBS three times and stored in a 1× PBS/96% EtOH (40 : 60) solution at −20°C (Schmidt & Eickhorst, 2014).

### Fluorescence *in situ* hybridisation and quantification

Samples from Lake Hampen, Lake Knud, Lake Cadagno and Aggersund were analysed by FISH following the protocol as described earlier (Pernthaler *et al*., 2001; Scholz *et al*., 2019).

The oligonucleotide probes DSB706 (Loy *et al*., 2002) labelled with Cy‐3 for the detection of filamentous Desulfobulbaceae and EUB‐MIX (Amann *et al*., 1990; Daims *et al*., 1999) labelled with Atto‐488 at a formamide concentration of 35% were used. The negative control probe NON‐EUB (Manz *et al*., 1992) labelled with Atto‐550 was run along on each multiwell slide. The samples were counterstained with 4′,6‐diamidino‐2‐phenylindole (DAPI; 1 mg ml^−1^) and the presence or absence of cable bacteria was determined using a fluorescence microscope. Cable bacteria in three replicates of the rhizosphere and surface samples from Aggersund were quantified by measuring the length of all positively stained filaments in each well using the imaging software NIS‐Elements (v.4.50; Nikon Instruments Inc., Melville, NY, USA) and the results were expressed as cable bacteria filament densities (m cm^−3^).

FISH and subsequent quantification of cable bacteria on the root segments (*c.* 0.5 cm) of either root tips or root hair zones of the *P*. *australis* seedlings grown in the rhizoboxes was carried out as described in Martin *et al*. (2018a). The probe Delta 495a‐c (Loy *et al*., 2002) with double‐labelled 6‐Fam for the detection of Deltaproteobacteria, the probes EUB‐MIX with double‐labelled Cy5 and the DSB706 probes labelled with Atto 565 at a formamide concentration of 35% were used. The NON‐EUB probe was labelled with Atto 565, 6‐Fam‐6‐Fam or Cy5‐Cy5. The root segments were then analysed by confocal laser scanning microscopy on a Nikon Ti‐E inverted microscope with a Nikon A1Si spectral detector. Manual counting of cells hybridised in each laser channel was performed using Imagej software and the Cell Counter plug‐in (https://imagej.nih.gov/ij/plugins/cell‐counter.html). To compensate for nonspecific binding, manual cell counting was also performed on the negative controls, and any cell numbers counted in the negative controls were subtracted from the corresponding sample counts. Each replicate was the average of 15 images and the results were expressed as cable bacteria cell densities (cells mm^−2^ root).

### DNA extraction and sequencing

DNA was extracted from root and bulk samples (total amount of samples: 42) from Lake Hampen, Lake Knud, Lake Cadagno, Aggersund and Arcachon using the DNeasy PowerLyzer PowerSoil Kit (Qiagen), according to the manufacturer’s protocol but with 60 µl instead of 100 µl elution buffer. Primers Bac341F (CCTACGGGNGGCWGCAG) and Bac805R (GACTACHVGGGTATCTAATCC) were used to amplify variable regions V3 and V4 of bacterial 16S rRNA genes (Herlemann *et al*., 2011). The 16S rRNA gene amplicon libraries were prepared according to Illumina’s 16S Metagenomic Sequencing Library Preparation guide with three consecutive PCR reactions for amplification of the target regions (1^st^ PCR: 20 cycles), addition of adapters (2^nd^ PCR: 10 cycles) and indexes (3^rd^ PCR: 8 cycles). Sequencing was carried out on a MiSeq desktop sequencer (Illumina, San Diego, CA, USA). The raw sequences can be downloaded from the NCBI/EMBL‐EBI/DDBJ Sequence Read Archive under BioProject ID ‘PRJNA680155’.

### Retrieval of public datasets

Criteria to choose public datasets for analysis were studies that involved next generation sequencing of the 16S rRNA gene of the microbial community in root‐associated or paddy soil samples. Raw sequences and metadata from 21 public datasets (Nguyen *et al*., 2015; Cúcio *et al*., 2016; Rothenberg *et al*., 2016; Shao *et al*., 2016; Ettinger *et al*., 2017; Fahimipour *et al*., 2017; Crump *et al*., 2018; Edwards *et al*., 2018; Gong *et al*., 2018; Kumar *et al*., 2018; Liu *et al*., 2018; Martin *et al*., 2019, 2018a, 2018b, 2020,2019, 2018a, 2018b, 2020; He *et al*., 2019; Hurtado‐McCormick *et al*., 2019; Lin *et al*., 2019; Ugarelli *et al*., 2019; Zhu *et al*., 2019; Ma *et al*., 2020) and two unpublished projects (Middleton *et al*., Ling *et al*.) including root and bulk samples of aquatic macrophytes (408 samples) and rice (1763 samples) were retrieved from the NCBI database and the Genome Sequence Archive (https://bigd.big.ac.cn/gsa/; Table S1).

A list of all samples analysed including the relevant metadata can be found in Table S2. To generalise the different sampling strategies used in each study, we termed ‘root samples’ to mean all samples that include roots and/or sediment that was washed off or sonicated free from roots in sterile buffer. ‘Bulk samples’ referred to root‐free zones except for samples from rice fields. Rice studies that reported actual root samples were Edwards *et al*. (2018) and Ma *et al*. (2020) (Table S2). Samples from all other rice studies were termed bulk samples as they were collected from the bulk paddy soil. However, these samples may have contained rhizosphere soil or root fragments.

The 16S rRNA dataset from Edwards *et al*. (2018) included 1510 samples from rice fields in California and Arkansas, USA that were taken throughout the growing season over 3 yr (2014–2016) consecutively. In this study, the rhizosphere was sampled from roots by vortexing the roots in PBS solution and collecting the slurry. The rhizoplane was sampled from cyclically washed and visibly clean roots by sonication and collecting the pellet after centrifugation. The endosphere fraction was achieved by grounding the washed and sonicated roots (in total 3 × for 30 s). Bulk samples were collected from a 0.5‐m‐wide walking lane in the rice field.

### Bioinformatic analysis

For both public and our newly generated data, primers were trimmed using cutadapt (v.1.16; Martin, 2011) and trimmed reads were processed using Dada2 (v.1.16.0, https://benjjneb.github.io/dada2/; Callahan *et al*., 2016). Taxonomy was assigned following the standard protocol of the DADA2 pipeline, modifying when necessary to accommodate single reads and to remove poor quality reads. The Ribosomal Database Project (RDP) classifier (Wang *et al*., 2007) and the SILVA SSURef NR 132 database (Quast *et al*., 2013) were used. The large dataset from Edwards *et al*. (2018) was divided into four groups that were run separately through the DADA2 pipeline and then merged into a full‐study sequence table before chimera removal using the ‘consensus’ method and taxa assignment. Sequences assigned as ‘Chloroplast’ and ‘Mitochondria’ were removed from each individual study. The assigned amplicon sequence variants (ASVs) were checked for the cable bacteria genera *Ca*. Electrothrix and *Ca*. Electronema. The data were analysed using RStudio (v.1.2.1335, RStudio Team), phyloseq (v.1.28.0; McMurdie & Holmes, 2013) and ggplot2 (v.3.2.1; Wickham, 2016).

Tentative species assignments were generated by performing a Blast (blastn v.2.2.29+; Camacho *et al*., 2009) search with the ASV sequences as queries against a database of representative full‐length cable bacteria 16S rRNA gene sequences (compiled from Trojan *et al*., 2016; Thorup, 2019). ASVs with an identity higher than 97% to a reference sequence across >90% of their length were assigned to that species. ASVs that were classified as *Ca*. Electrothrix or Electronema by Dada2 but lacked identity above this threshold to reference sequences are listed as unidentified. A phylogenetic tree was constructed from reference sequences using the Sina aligner v.1.2.11 (Pruesse *et al*., 2012) and Phyml v.3.1 (Guindon & Gascuel, 2003) software with 1000 bootstraps.

### Statistics

The following analyses were carried out in R (v.3.6.0, R Core Team, 2013). Differences in variances of the cable bacteria density between the sampling regions of the FISH samples from Aggersund and the rhizobox experiment were tested using an *F*‐test. The variances of the cable bacteria filament densities between rhizosphere and surface sediment in samples from Aggersund were equal (significance level α = 0.05). Therefore, the differences of the means were tested using unpaired two‐tailed Student’s *t*‐test with a significance level of 0.05, six observations and four degrees of freedom. For the rhizobox experiment, variances of the cable bacteria cell density between the root hair region and the root tip, elongation and immature root region were unequal. Therefore, a Welch test was carried out to test the differences of the means between the root regions with a significance level of 0.05 and six observations. The degrees of freedom were 2, 2.0056 and 2.0451, respectively. Differences for the fractions of samples with cable bacteria per month in the rice fields were tested using one‐way ANOVA.

## Results

### Breadth of the plant–cable bacteria relationship

Cable bacteria were found by 16S rRNA sequencing roots, rhizosphere or paddy soil in 36 out of 55 investigated sites around the world and encompassing four of the five investigated freshwater plant species, 10 of the 14 investigated seagrass species and one of the eight saltmarsh plants. Cable bacteria were present in rice fields in eight out of 13 investigated sites (Fig. 1; Table S1).

**Fig. 2:**
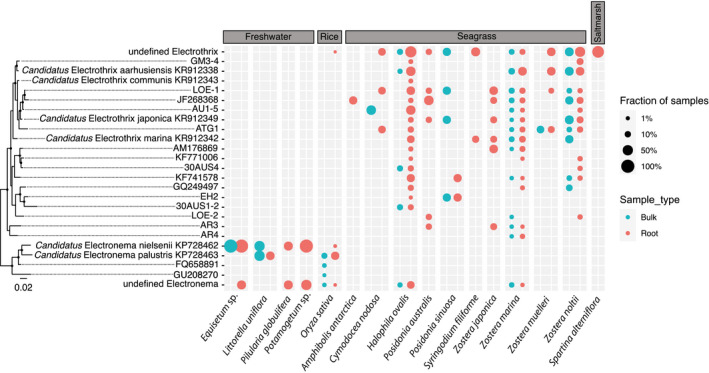
Phylogenetic diversity and fraction of cable bacteria species. The fraction represents the amount of samples that contain the specific cable bacteria species over the total amount of root or bulk samples per plant species. Root samples include samples from the rhizosphere, rhizoplane or endosphere. Bulk samples were collected from root‐free zones or paddy soils. The absence of cable bacteria in bulk samples from certain species indicates that cable bacteria were either absent in the bulk samples or that no bulk samples were analysed (this is evident in Fig. 3). Reference 16S rRNA gene sequences without an accession number (GM3‐4, LOE‐1, AU1‐5, ATG1, 30AUS4, EH2, 30AUS1‐2, LOE‐2, AR3, AR4) are from Thorup (2019). Tree nodes with black points indicate bootstrap values greater than 50%. The outgroup sequence forming the root of the tree (not displayed) was AY548789.1 (*Desulfobulbus propionicus*).

In Lake Knud, the two described freshwater cable bacteria species, *Ca*. Electronema palustris and *Ca*. Electronema nielsenii, and undefined Electronema species were identified around roots of *L*. *uniflora* and *P*. *globulifera* (Fig. 2). Both known cable bacteria species were also present in the surface sediment close to the *L*. *uniflora* meadow.

By contrast, *Ca*. Electronema nielsenii and the undefined Electronema species occurred in Lake Cadagno around the roots of *Equisetum* sp. and *Potamogeton* sp. (Fig. 2). Cable bacteria associated with rice and paddy soil included both known *Ca*. Electronema species, and two sequences classified as *Ca*. Electronema in the SILVA database (accession nos. GU208270 and FQ658891), as well as undefined Electronema and Electrothrix species.

Cable bacteria around seagrass and associated bulk samples were more diverse than in the freshwater environments and included all four described *Ca*. Electrothrix species, 15 newly proposed species (Thorup, 2019), as well as unclassified Electrothrix species. Cable bacteria diversity was higher in root samples compared with the bulk samples (16 vs 3 defined species) for *H*. *ovalis*. However, this trend was not observed in samples from *Z. marina* and *Z*. *noltii*, which showed high diversity in both root and bulk samples. Interestingly, undefined Electronema species appeared in root and bulk samples of *H*. *ovalis* and *Z. marina*.

Moreover, only undefined cable bacteria species were found around roots of the saltmarsh plant *Spartina alterniflora*. Taken together, no specific cable bacteria species appeared to dominate in the root or bulk samples of the freshwater, terrestrial and marine environments.

Thick and thin cable bacteria filaments were found on seagrass roots, with cable filament widths of around 4 µm and 1 µm on roots of *P*. *australis* seedlings and filament widths of around 6 µm and 1 µm in the rhizosphere of *Z. marina* (Fig. 1, FISH images).

**Fig. 3:**
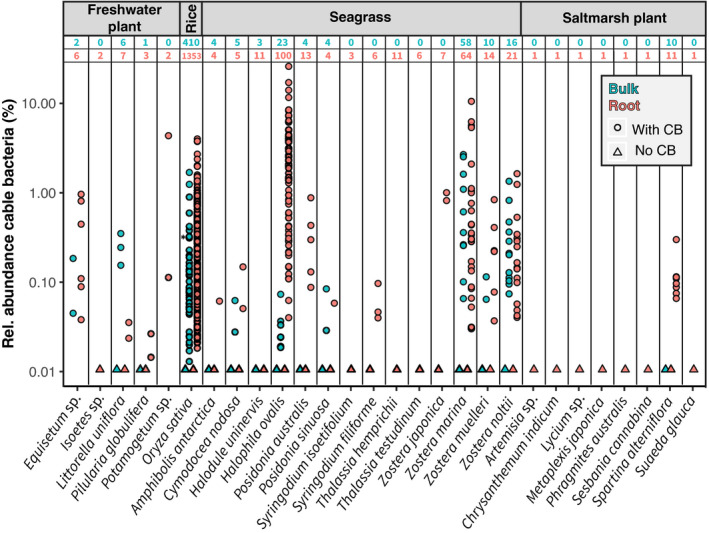
Relative abundance of plant‐associated cable bacteria (from 16S rRNA gene data). The relative cable bacteria abundance is plotted as a function of plant and sample type. For better presentation of the zero values, 0.01 was added to all samples. Note the logarithmic scale on the *y*‐axis. Blue and red numbers refer to the total amount of analysed bulk and root samples, respectively. Root samples include samples from the rhizosphere, rhizoplane or endosphere. For *Oryza sativa*, all root samples with cable bacteria were collected from rice fields in the USA. Bulk samples were collected from root‐free zones or paddy soil. The asterisk indicates the sample with the highest relative abundance of cable bacteria in paddy soil in Asia. Note that the samples from *Posidonia* *australis* were retrieved from the field (Supporting Information Tables S1, S2).

In the oligotrophic Lake Hampen no cable bacteria were found associated with roots of *L*. *uniflora* nor with the bulk sediment by means of FISH and 16S rRNA gene sequencing (Fig. S2). In Lake Knud, cable bacteria were found in association with the roots of *L*. *uniflora* and *P*. *globulifera*, as well as in the bulk sediment. The maximum relative abundance of cable bacteria in the root microbiome of both plants was 0.02% and 0.01%. (Fig. 3).

In the meromictic Lake Cadagno, cable bacteria were present around roots of *Equisetum* sp. and *Potamogeton* sp. and the highest relative abundances of cable bacteria in the root microbiomes were 0.9% and 4.07%, respectively. By contrast, roots of *Isoetes* sp. that were sampled from the wetland in immediate vicinity of Lake Cadagno, did not show cable bacteria by FISH or 16S rRNA gene sequencing (Fig. S2). Cable bacteria relative abundances appeared to be higher around the roots of the fast‐growing, seagrass species *Zostera* spp. and *Halophila ovalis* compared with other seagrass species. Cable bacteria relative abundances tended also to be higher on the roots compared with the bulk samples, particularly for *H*. *ovalis*. However, it was difficult to compare abundance information from 16S rRNA gene sequencing across studies and we therefore specifically quantified the cable bacteria abundances in the rhizosphere and at the surface bulk for *Z. marina* in Aggersund by FISH (see section below). Cable bacteria were also found in both tropical (*Syringodium filiforme* and *Cymodocea nodosa*) and temperate seagrass species (*Posidonia* spp. and *Amphibolis* *antarctica*; Fig. 3).

In the marine strait Aggersund (Fig. 1), cable bacteria were found in the root samples of *Z. marina* with a maximum relative abundance of 9.85%. The maximum relative abundance of all samples was found in a root sample of *H*. *ovalis* from Pelican Point, Australia (dataset: J.A. Middleton *et al*., unpublished) and was 24.21%.

Only 0.02% (12 reads total) of *Ca*. Electrothrix were found in one out of three root samples from *Z*. *noltii* in Arcachon, France by 16S rRNA sequencing. In comparison, the other seagrass samples from our field study showed 2–10% (1180–4574 reads total) cable bacteria for *Z. marina* from Aggersund in three out of four samples. Furthermore, the *Ca*. Electrothrix species of the sample from *Z*. *noltii* in Arcachon could not be confirmed by the Blast search. This combination of low abundance, a single positive sample, and poor sequence identity to known cable bacteria have led to the conservative judgement not to include *Z*. *noltii* roots from Arcachon in our overview of samples with cable bacteria present (Fig. 1).

The highest relative abundance of cable bacteria around rice roots was 3.73% found in the USA (Figs 3, S3). Cable bacteria were also found associated with paddy soil in China, India and Vietnam (Fig. 1; Table S1) when the maximum relative abundance of cable bacteria was 0.29% (see asterisk in Fig. 3).

### Cable bacteria distribution and dynamics in rice fields

**Fig. 4:**
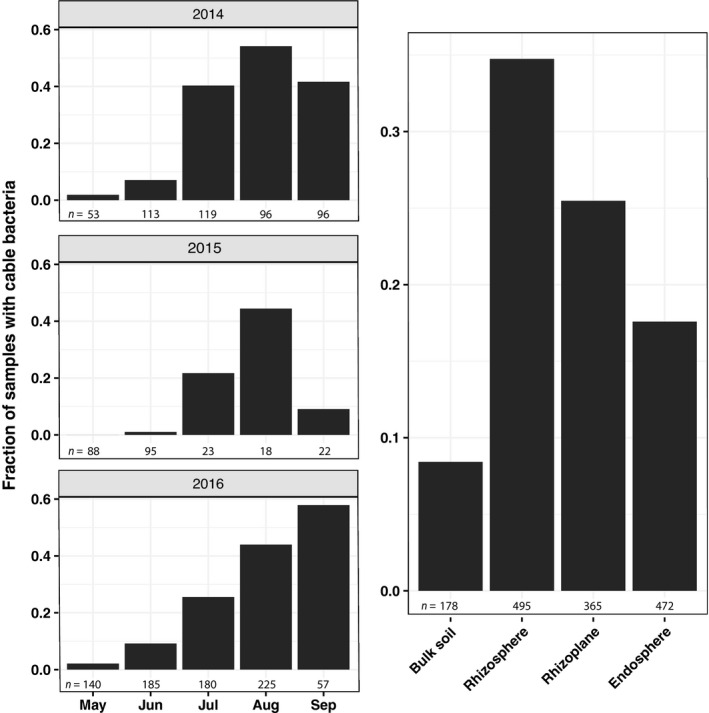
Fraction of samples with rice‐associated cable bacteria (from 16S rRNA gene data). Fraction of total samples with cable bacteria during the growing season from May to September over 3 yr (left three panels). Fraction of samples with cable bacteria across the root compartments and bulk soil (right panel). All graphs are based on data from Edwards *et al*. (2018). Numbers refer to the total amount of samples in each month and compartment.

The re‐analysis of the published 16S rRNA gene dataset from rice fields in California and Arkansas, USA (Edwards *et al*., 2018), indicates that the cable bacteria population followed similar growth patterns interannually throughout the growing seasons, which started in May when fields were flooded. From plotting the relative abundances of cable bacteria it appears that cable bacteria were less abundant in the months May to June compared with July to September (Fig. S3). However, this trend was strongly influenced by the high number of zero values. To unravel a clearer succession of the cable bacteria population, relative abundance was reduced to the presence or absence of cable bacteria and reported as the fraction of samples that contained at least one read of cable bacteria. The fraction of samples with cable bacteria increased from May to August in all years. The increasing fraction implies the spread of a dense localised population to a wider area and/or the growth of cable bacteria over time to densities above the detection limit of the extraction and sequencing of the cable bacteria 16S rRNA gene. In September, the fraction dropped in the years 2014 and 2015, but further increased in 2016 (Fig. 4). The differences of cable bacteria fractions between the months of the growing season were significant (one‐way ANOVA; *F*(4,10) = 7.711, *P* < 0.01). Furthermore, the fraction of samples with cable bacteria decreased from the rhizosphere to the endosphere compartment. The bulk soil showed the lowest fraction of cable bacteria‐positive samples (Fig. 4).

### Occurrence of cable bacteria in seagrass seedlings (*Posidonia australis*) and spatial organisation around roots

**Fig. 5:**
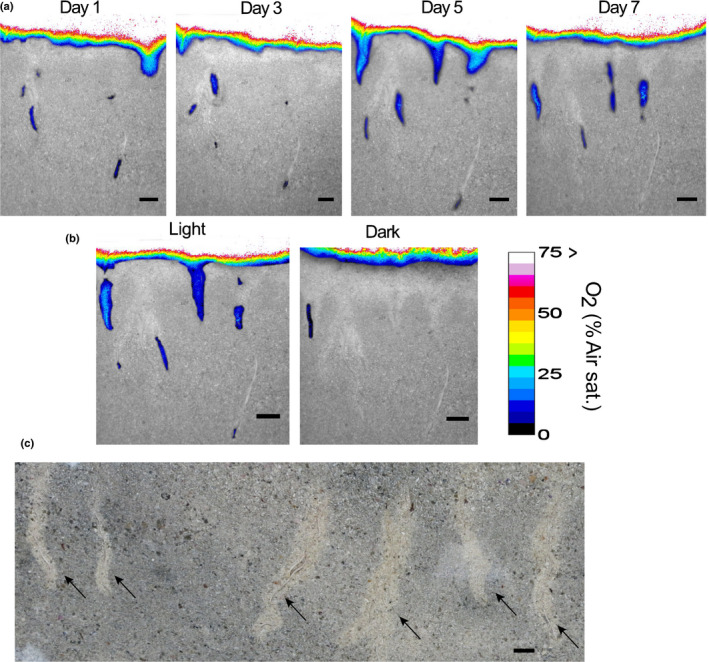
Oxygen loss from roots of *Posidonia* *australis* seedlings. (a) Oxygen loss from roots over 8 d of growth (each panel 2 d apart). (b) Oxygen loss from roots during light (250 µmol photons m^−2^ s^−1^) and dark transitions (30 min darkness). Images are overlays of transparent optode images on black‐and‐white images of the roots. (c) Photograph of roots surrounded by oxidised sediment (yellow‐coloured sediment indicated by black arrows). Bars, 5 mm. Oxygen concentrations are given as the unit % air saturation.

The rhizobox experiment showed that newly emerged roots of *P*. *australis* had the greatest oxygen release, compared with older roots. Oxygen was also leaking from newly developing shoots or rhizomes at day 1 and day 5 of the incubation experiment (Fig. 5). The growing, leaking root segments were on average 10 ± 1 mm long (mean ± SE, *n* = 3) and the maximum oxygen concentration was 26 ± 3% (mean ± SE, *n* = 3) air saturation (Table S3). When the light was turned off, oxygen around most of the roots was consumed within 30 min (Fig. 5b).

**Fig. 6:**
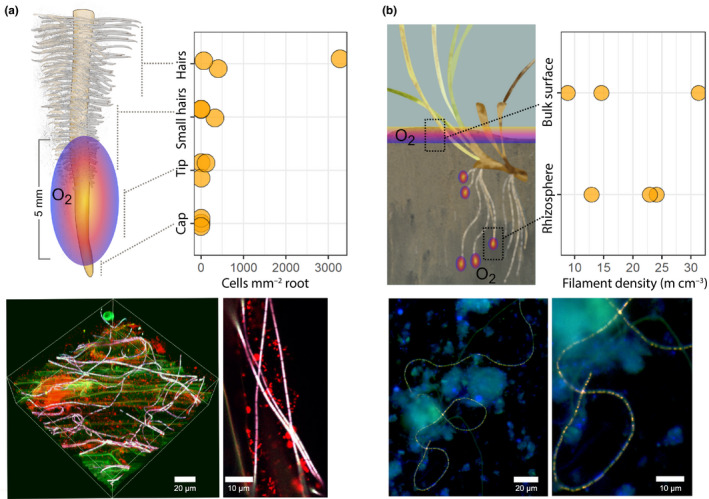
Cable bacteria distribution around seagrass roots. (a) Fluorescence *in situ* hybridisation (FISH) cell counts of cable bacteria on the surface of root zones of *Posidonia* *australis* seedlings. FISH image shows hybridisations with the probes Delta 495a‐c specific for Deltaproteobacteria (green), EUB‐MIX targeting most bacteria (red) and DSB706, specific for Desulfobulbaceae (blue). Cable bacteria appear purple/whitish from overlay of all three probes, other bacteria are red, and the surface of the root can be seen as green autofluorescence. (b) Cable bacteria filament densities in the rhizosphere of *Zostera marina* and top 2 cm surface sediment. FISH images show hybridisations with probes DSB706 (red) and EUB‐MIX (green), counterstained with DAPI (blue). Cable bacteria appear yellow from overlay of the two probes, while other cells (and DNA in cable bacteria) appear blue.

The cable bacteria cell density appeared to decrease from the root hair zone (1250 ± 1020 cells mm^−2^, mean ± SE, *n* = 3) to the zone of small, newly developed root hairs (100 ± 100 cells mm^−2^, mean ± SE, *n* = 3) and the root tip (40 ± 40 cells mm^−2^, mean ± SE, *n* = 3; Fig. 6a). No cable bacteria cells were found on the root cap. However, the abundance in the root hair zone was not significantly different compared with the ‘small root hair zone’ (*P* = 0.38, *n* = 6, Welch test), the root tip (*P* = 0.36, *n* = 6, Welch test) and root cap (*P* = 0.34, *n* = 6, Welch test). Moreover, Deltaproteobacteria followed a similar distribution and abundance to cable bacteria. By contrast, the counts of all bacteria showed no clear partitioning along the roots (Fig. S4).

The cable bacteria filament density in the oxic/anoxic transition zone in the rhizosphere of *Z. marina* (20 ± 4 m cm^−3^, mean ± SE, *n* = 3) was as high as the one found in the oxic/anoxic transition zone of the bulk surface sediment (20 ± 7 m cm^−3^, mean ± SE, *n* = 3; *P* = 0.83, *n* = 6, unpaired two‐tailed *t*‐test; Fig. 6b).

## Discussion

### Diversity

Our study expanded the known habitats of cable bacteria by reporting the *in situ* occurrence of cable bacteria around roots of various plants in a diverse set of environments, including freshwater and marine environments, as well as rice fields. The breadth of this relationship across different plant types and ecosystems, as well as the high diversity of cable bacteria associated with the roots, bulk sediments and paddy soils (Figs 1–3; Table S1), showed no evidence for species‐specific associations between plants and cable bacteria. All six described candidate cable bacteria species, which have been found in various nonvegetated locations (Trojan *et al*., 2016), can also grow around plant roots (Fig. 2). Overall, no cable bacteria species, including the proposed and undefined candidate species, was found to be exclusively root specific.

The high cable bacteria diversity around seagrass roots appeared to match our observation of two different cable bacteria morphologies co‐existing around seagrass roots. The thick and thin filaments could be genotypic features of different marine cable bacteria species. Interestingly, around roots of *H*. *ovalis* and *Z. marina*, undefined Electronema species were detected that would be usually expected to be found in freshwater environments (Trojan *et al*., 2016; Dam *et al*., 2021). Groundwater inflow might locally decrease the salinity and create site‐specific habitats when freshwater and marine cable bacteria could co‐exist. Alternatively, these undefined Electronema species may tolerate salinity.

### Drivers

The comparisons of the relative abundances of root and bulk samples from the 16S rRNA gene data indicated higher relative abundances of cable bacteria at the roots of various plant species, including *H*. *ovalis* and *S*. *alterniflora* (Fig. 3). However, the analysed public datasets included root and bulk samples that did not specifically target oxygen‐releasing plant roots and the top 2 cm surface sediment – the sites at which the most of the cable bacteria cells would be expected. Therefore, the data from the public datasets may underestimate the relative abundance of cable bacteria associated with roots of aquatic plants and in bulk samples.

Furthermore, we quantified cable bacteria in the rhizosphere of *Z. marina* and at the oxic–anoxic interface at the sediment surface. The results revealed similar absolute abundances of cable bacteria around the roots of *Z. marina* and at the top surface sediment (Fig. 6b), which underlined the idea that the rhizosphere is a true expansion of the classical cable bacteria habitat. Typically, cable bacteria have been found at oxic–anoxic interfaces of sediment surfaces where they reduce the oxygen from the water column. Our results, in combination with recent studies that showed cable bacteria around worm and shrimp burrows (Aller *et al*., 2019; Li *et al*., 2020), extend the cable bacteria habitat to a 3D grid deeper down into the sediments and soils.

Roots of seagrass seedlings, *P*. *australis,* oxygenated the rhizosphere at the growing root tip (Fig. 5) whereas the root hair region did not leak oxygen. This pattern of rhizosphere oxygenation has previously only been shown for adult seagrass species (Jovanovic *et al*., 2015; Martin *et al*., 2018). However, cable bacteria were more abundant in the root hair region than at the oxygen‐releasing tips of *P*. *australis* (Fig. 6a), which was also observed for roots of the seagrasses *Z*. *muelleri* and *H*. *ovalis* (Martin *et al*., 2018a). The observed higher cable bacteria cell abundance in the root hair region could be explained in two ways. First, cable bacteria grow along the root, with one end in the oxygenated top sediment layer or in the oxygenated rhizosphere around the root tip region. Here, the latter is supported by the observations that cable bacteria can adjust their position (Malkin & Meysman, 2015) with a speed of 2.9 mm h^−1^ (Bjerg *et al*., 2016). This rate of movement would be sufficiently fast to trace the downward growing, oxygen‐releasing, root tip. The sulfide‐oxidising cable bacteria cells located along the root surface could be stimulated by root exudates feeding sulfate reduction in the root hair region. Second, the cable bacteria may penetrate the root to directly tap into the oxygen‐rich aerenchyma, while the other end consumes sulfide on the outside of the root. This second explanation would qualify the parts of cable bacteria filaments embedded in the roots as endophytes. Indeed, the re‐analysis of the 16S rRNA gene dataset from Edwards *et al*. (2018) indicated cable bacteria cells in the inside of rice roots (Fig. 4). Endophytic bacteria are known to penetrate the epidermis at the site of lateral root extensions (Dong *et al*., 2003), which agrees with observations from Scholz *et al*. (2019), showing cable bacteria running along root hairs pointing towards the root surface. However, the rice root samples were separated into the root compartments (rhizosphere, rhizoplane and endosphere) by washing and sonication procedures and subsequently analysed by 16S rRNA gene sequencing (Edwards *et al*., 2018). Therefore, sampling artefacts cannot be entirely excluded and microscopic investigations are needed to reveal if cable bacteria are true endophytes. In rice, the cable bacteria sample fraction decreased from the rhizosphere to the endosphere compartment (Fig. 4). The observations that the abundance of cable bacteria cells decreased at the sites where cable bacteria would source the oxygen (endosphere compared with the outside of rice root; at the root tip of seagrass compared with the root hair region) is in accordance with observations from cable bacteria in nonvegetated sediments. In these sediments less than one‐tenth of each filament is in the oxic zone (Scilipoti *et al*., 2021) and most of the cable bacteria cells are located in the reduced sediment layer below the oxic surface (Schauer *et al*., 2014; van de Velde *et al*., 2016).

In addition to the microscale oxygen availability, we found that sulfide availability in the environment is also likely to control the abundance of cable bacteria. No cable bacteria were found to be associated with *L*. *uniflora* in the oligotrophic Lake Hampen, but their presence was confirmed in the mesotrophic Lake Knud. The relative abundance of plant‐associated cable bacteria was higher in sulfide‐rich Lake Cadagno sediment. The similar relative abundance of cable bacteria around rice roots could be explained by the generally high turnover of organic matter in rice fields that stimulates sulfate reduction (Wind & Conrad, 1997). The highest relative abundance of plant‐associated cable bacteria was recorded for seagrass, where high amounts of sulfide ensure a continuous source of substrate for cable bacteria. More detailed sulfide data are needed to disentangle how the availability of electron donor sources (free sulfide vs iron sulfide) for cable bacteria influences their abundance and activity. However, the absence or near absence of cable bacteria around roots in sulfide‐rich sediments, for example *Isoetes* sp. at Lake Cadagno or *Z*. *noltii* in Arachon, France, indicates that the establishment of a plant–cable bacteria relationship is also determined by factors other than oxygen and sulfide availability.

The annual re‐establishment of cable bacteria in rice fields after flooding (Fig. 4) as well as the presence of cable bacteria on roots in the pristine, high alpine Lake Cadagno (Fig. 1), show that cable bacteria populations are robust and not limited to open systems such as marine environments (Burdorf *et al*., 2017) and freshwater streams (Risgaard‐Petersen *et al*., 2015).

### Ecological consequences

From our synthesis we showed that cable bacteria form a generic member of root microbiomes of aquatic plants across a diversity of ecosystems. However, we found that cable bacteria were not associated with all roots (e.g. cable bacteria were absent in 65% of the rhizosphere samples from rice fields in the USA; Fig. 4) and that for instance, *L*. *uniflora* can live with and without cable bacteria (Figs 1, S2). The negative findings and the high diversity of plant‐associated cable bacteria indicated that the association is not obligatory for either of the partners. But the positive findings with cable bacteria densities as high as in the bulk surface sediment, where their impact on geochemical cycling is recognised (Risgaard‐Petersen *et al*., 2012; Seitaj *et al*., 2015), make further investigations into their potential impact on plant health worthwhile. For example, the cable bacteria–plant relationship could alleviate critical sulfide toxicity in seagrass meadows (Borum *et al*., 2005) and rice fields (Joshi *et al*., 1975). Furthermore, electrogenic sulfide oxidation can lead to acidification by 1 pH unit (Burdorf *et al*., 2018; Sandfeld *et al*., 2020), which may mobilise the essential plant nutrients iron and phosphorous (Brodersen *et al*., 2017). By contrast, excess iron is toxic for plants (Mehraban *et al*., 2008) and the potential cable bacteria‐driven mobilisation of trace metals, such as arsenic in contaminated Asian rice fields, may result in increased levels of arsenic in rice grain and arsenic‐related human diseases upon intake (Meharg, 2004; van de Velde *et al*., 2017). However, cable bacteria may also promote the formation of iron oxide–hydroxide plaques on oxygen‐releasing plant roots, which would be a firewall against toxic sulfide and arsenic uptake into roots, but also be a sink for phosphorus (Liu *et al*., 2004; Seitaj *et al*., 2015; Sulu‐Gambari *et al*., 2016; van de Velde *et al*., 2017). Furthermore, cable bacteria may increase nitrogen availability for the plants as they stimulate dissimilatory nitrate reduction to ammonium (Kessler *et al*., 2019) and have the potential to fix N_2_ (Kjeldsen *et al*., 2019). These biogeochemical effects of cable bacteria around the roots of aquatic plants and rice, which are likely to change on a diurnal basis, should be studied for the different plant, sediment and soil types.

The cable bacteria‐mediated impact on plant health could be particularly important for seedling vigour. Here, we show for the first time both oxygen loss and cable bacteria on a *Posidonia* species and seedlings. This suggests a close association throughout the life of seagrasses and lays the groundwork for potential improvements in seed‐based seagrass restoration techniques. Seagrass meadows are in decline across the globe, which has subsequently spearheaded significant investment in seagrass restoration. However, restoration of marine ecosystems is still considered a developing area of science and is associated with a relatively high cost compared with terrestrial restoration (Tan *et al*., 2020). In this context, roots precoated with naturally enriched cable bacteria could be a low‐cost solution to increase seedling vigour upon transplantation.

Furthermore, inoculation of cable bacteria in rice pots has been found to reduce methane emissions (Scholz *et al*., 2020). We show that cable bacteria grow in rice fields in Asia and the USA, which expands the known habitats of cable bacteria. Not only were cable bacteria found to be closely associated with rice roots (rhizosphere, rhizoplane and endosphere) in the USA, but their occurrence was also linked to the annual growing season (Fig. 4). It is vital to better understand the drivers behind the repeated peak abundances in the second half of the growing season. By contrast with rice fields in the USA, which usually remain continuously flooded throughout the growing season, common practices in Asia include the intermittent drainage of rice fields. The question whether the short‐term aerations of the paddy soil would impede the establishment of peak abundances, as observed for rice fields in the USA, still remains open. Understanding the cable bacteria dynamics in rice fields and their impact on plant health, as well as metal mobilisation (e.g. arsenic), will help to develop novel management practices to stimulate continuous high cable bacteria abundances throughout the entire growing season. These practices will, therefore, potentially reduce emissions of the potent greenhouse gas methane, while avoiding negative side effects.

## Author contributions

VVS, BCM, MWF, AS, LPN, GAK, NRP and IPGM conceived, designed and planned the work. VVS, BCM, RM, AS, MWF and LDWB carried out the field work. VVS, BCM, MWF and RM carried out FISH experiments. VVS and BCM carried out 16S rRNA gene sequencing. VVS, BCM, IPGM analysed the 16S rRNA gene sequencing data. BCM and MWF carried out the planar optode measurements and analysis. All authors contributed to the interpretation of the data and production of the manuscript.

## Supporting information


**Fig. S1** Collection of *Posidonia australis* seeds and rhizobox design.
**Fig. S2** Photographs of sample locations without abundant plant‐associated cable bacteria.
**Fig. S3** Relative abundance of cable bacteria in rice fields (from 16S rRNA gene data).
**Fig. S4** Abundance of all bacteria, cable bacteria and Deltaproteobacteria along roots of *P*. *australis* seedlings.

**Table S1** Overview of all 16S rRNA gene datasets.
**Table S2** Overview of all 16S rRNA gene samples analysed.
**Table S3** Oxygen loss from *Posidonia australis* roots during light and dark.
Please note: Wiley Blackwell are not responsible for the content or functionality of any Supporting Information supplied by the authors. Any queries (other than missing material) should be directed to the *New* *Phytologist* Central Office.

## Data Availability

The data that support the findings of this study are available from the corresponding author upon reasonable request. Raw 16S rRNA gene sequences can be downloaded from the NCBI database (see Table S1 for references and accession numbers).

## References

[nph17415-bib-0001] Aller RC , Aller JY , Zhu Q , Heilbrun C , Klingensmith I , Kaushik A . 2019. Worm tubes as conduits for the electrogenic microbial grid in marine sediments. Science Advances 5: eaaw3651. 31328163 10.1126/sciadv.aaw3651 PMC6636988

[nph17415-bib-0002] Amann RI , Binder BJ , Olson RJ , Chisholm SW , Devereux R , Stahl DA . 1990. Combination of 16S rRNA‐targeted oligonucleotide probes with flow cytometry for analyzing mixed microbial populations. Applied and Environmental Microbiology 56: 1919–1925. 2200342 10.1128/aem.56.6.1919-1925.1990 PMC184531

[nph17415-bib-0003] Bálint M , Bahram M , Eren AM , Faust K , Fuhrman JA , Lindahl B , O'Hara RB , Öpik M , Sogin ML , Unterseher M *et al*. 2016. Millions of reads, thousands of taxa: microbial community structure and associations analyzed via marker genes. FEMS Microbiology Reviews 40: 686–700. 27358393 10.1093/femsre/fuw017

[nph17415-bib-0004] Berendsen RL , Pieterse CMJ , Bakker PAHM . 2012. The rhizosphere microbiome and plant health. Trends in Plant Science 17: 478–486. 22564542 10.1016/j.tplants.2012.04.001

[nph17415-bib-0005] Bjerg JT , Damgaard LR , Holm SA , Schramm A , Nielsen LP . 2016. Motility of electric cable bacteria. Applied and Environmental Microbiology 82: 3816–3821. 27084019 10.1128/AEM.01038-16 PMC4907201

[nph17415-bib-0006] Borum J , Pedersen O , Greve T , Frankovich T , Zieman J , Fourqurean JW , Madden C . 2005. The potential role of plant oxygen and sulphide dynamics in die‐off events of the tropical seagrass, *Thalassia testudinum* . Journal of Ecology 93: 148–158.

[nph17415-bib-0007] Brodersen KE , Koren K , Moßhammer M , Ralph PJ , Kühl M , Santner J . 2017. Seagrass‐mediated phosphorus and iron solubilization in tropical sediments. Environmental Science & Technology 51: 14155–14163. 29149570 10.1021/acs.est.7b03878 PMC5738630

[nph17415-bib-0008] Brodersen KE , Nielsen DA , Ralph PJ , Kühl M . 2015. Oxic microshield and local pH enhancement protects *Zostera muelleri* from sediment derived hydrogen sulphide. New Phytologist 205: 1264–1276. 10.1111/nph.13124 25367685

[nph17415-bib-0009] Burdorf LD , Hidalgo‐Martinez S , Cook PL , Meysman FJ . 2016. Long‐distance electron transport by cable bacteria in mangrove sediments. Marine Ecology Progress Series 545: 1–8.

[nph17415-bib-0010] Burdorf LD , Malkin SY , Bjerg JT , van Rijswijk P , Criens F , Tramper A , Meysman FJ . 2018. The effect of oxygen availability on long‐distance electron transport in marine sediments. Limnology and Oceanography 63: 1799–1816.

[nph17415-bib-0011] Burdorf LDW , Tramper A , Seitaj D , Meire L , Hidalgo‐Martinez S , Zetsche EM , Boschker HTS , Meysman FJR . 2017. Long‐distance electron transport occurs globally in marine sediments. Biogeosciences 14: 683–701.

[nph17415-bib-0012] Callahan BJ , McMurdie PJ , Rosen MJ , Han AW , Johnson AJA , Holmes SP . 2016. DADA2: high‐resolution sample inference from Illumina amplicon data. Nature Methods 13: 581–583. 27214047 10.1038/nmeth.3869 PMC4927377

[nph17415-bib-0013] Camacho C , Coulouris G , Avagyan V , Ma N , Papadopoulos J , Bealer K , Madden TL . 2009. BLAST+: architecture and applications. BMC Bioinformatics 10: 421. 20003500 10.1186/1471-2105-10-421 PMC2803857

[nph17415-bib-0014] Christensen PB , Sørensen J . 1986. Temporal variation of denitrification activity in plant‐covered, littoral sediment from Lake Hampen, Denmark. Applied and Environmental Microbiology 51: 1174–1179. 16347074 10.1128/aem.51.6.1174-1179.1986 PMC239041

[nph17415-bib-0015] Crump BC , Wojahn JM , Tomas F , Mueller RS . 2018. Metatranscriptomics and amplicon sequencing reveal mutualisms in seagrass microbiomes. Frontiers in Microbiology 9: 388. 29599758 10.3389/fmicb.2018.00388 PMC5863793

[nph17415-bib-0016] Cúcio C , Engelen AH , Costa R , Muyzer G . 2016. Rhizosphere microbiomes of European seagrasses are selected by the plant, but are not species specific. Frontiers in Microbiology 7: 440. 27065991 10.3389/fmicb.2016.00440 PMC4815253

[nph17415-bib-0017] Daims H , Brühl A , Amann R , Schleifer K‐H , Wagner M . 1999. The domain‐specific probe EUB338 is insufficient for the detection of all bacteria: development and evaluation of a more comprehensive probe set. Systematic and Applied Microbiology 22: 434–444. 10553296 10.1016/S0723-2020(99)80053-8

[nph17415-bib-0018] Dam A‐S , Marshall IPG , Risgaard‐Petersen N , Burdorf LDW , Marzocchi U . 2021. Effect of salinity on cable bacteria species composition and diversity. Environmental Microbiology 23: 2605–2616. 33760391 10.1111/1462-2920.15484 PMC8252435

[nph17415-bib-0019] Dong Y , Iniguez AL , Triplett EW . 2003. Quantitative assessments of the host range and strain specificity of endophytic colonization by *Klebsiella pneumoniae* 342. Plant and Soil 257: 49–59.

[nph17415-bib-0020] Edwards JA , Santos‐Medellín CM , Liechty ZS , Nguyen B , Lurie E , Eason S , Phillips G , Sundaresan V . 2018. Compositional shifts in root‐associated bacterial and archaeal microbiota track the plant life cycle in field‐grown rice. PLoS Biology 16: e2003862. 29474469 10.1371/journal.pbio.2003862 PMC5841827

[nph17415-bib-0021] Erftemeijer PL , Koch EW . 2001. Sediment geology methods for seagrass habitat. Amsterdam, the Netherlands: Elsevier Science.

[nph17415-bib-0022] Ettinger CL , Voerman SE , Lang JM , Stachowicz JJ , Eisen JA . 2017. Microbial communities in sediment from *Zostera marina* patches, but not the *Z. marina* leaf or root microbiomes, vary in relation to distance from patch edge. PeerJ 5: e3246. 28462046 10.7717/peerj.3246 PMC5410140

[nph17415-bib-0023] Fahimipour AK , Kardish MR , Lang JM , Green JL , Eisen JA , Stachowicz JJ . 2017. Global‐scale structure of the eelgrass microbiome. Applied and Environmental Microbiology 83: e3391–e3416. 10.1128/AEM.03391-16 PMC5452814 28411219

[nph17415-bib-0024] Gaiero JR , McCall CA , Thompson KA , Day NJ , Best AS , Dunfield KE . 2013. Inside the root microbiome: bacterial root endophytes and plant growth promotion. American Journal of Botany 100: 1738–1750. 23935113 10.3732/ajb.1200572

[nph17415-bib-0025] Gong Y , Bai J‐L , Yang H‐T , Zhang W‐D , Xiong Y‐W , Ding P , Qin S . 2018. Phylogenetic diversity and investigation of plant growth‐promoting traits of actinobacteria in coastal salt marsh plant rhizospheres from Jiangsu, China. Systematic and Applied Microbiology 41: 516–527. 29934111 10.1016/j.syapm.2018.06.003

[nph17415-bib-0026] Guindon S , Gascuel O . 2003. A simple, fast, and accurate algorithm to estimate large phylogenies by maximum likelihood. Systematic Biology 52: 696–704. 14530136 10.1080/10635150390235520

[nph17415-bib-0027] He M , Zhang J , Shen L , Xu L , Luo W , Li D , Zhai N , Zhao J , Long Y , Pei X *et al*. 2019. High‐throughput sequencing analysis of microbial community diversity in response to *indica* and *japonica* bar‐transgenic rice paddy soils. PLoS ONE 14: e0222191. 31498816 10.1371/journal.pone.0222191 PMC6733487

[nph17415-bib-0028] Herlemann DPR , Labrenz M , Jürgens K , Bertilsson S , Waniek JJ , Andersson AF . 2011. Transitions in bacterial communities along the 2000 km salinity gradient of the Baltic Sea. The ISME Journal 5: 1571–1579. 21472016 10.1038/ismej.2011.41 PMC3176514

[nph17415-bib-0029] Hurtado‐McCormick V , Kahlke T , Petrou K , Jeffries T , Ralph PJ , Seymour JR . 2019. Regional and microenvironmental scale characterization of the *Zostera muelleri* seagrass microbiome. Frontiers in Microbiology 10: 1011. 31139163 10.3389/fmicb.2019.01011 PMC6527750

[nph17415-bib-0030] Joshi M , Ibrahim I , Hollis J . 1975. Hydrogen sulfide: effects on the physiology of rice plants and relation to straighthead disease. Phytopathology 65: 1165–1170.

[nph17415-bib-0031] Jovanovic Z , Pedersen MØ , Larsen M , Kristensen E , Glud RN . 2015. Rhizosphere O_2_ dynamics in young *Zostera marina* and *Ruppia maritima* . Marine Ecology Progress Series 518: 95–105.

[nph17415-bib-0032] Kessler AJ , Wawryk M , Marzocchi U , Roberts KL , Wong WW , Risgaard‐Petersen N , Meysman FJ , Glud RN , Cook PL . 2019. Cable bacteria promote DNRA through iron sulfide dissolution. Limnology and Oceanography 64: 1228–1238.

[nph17415-bib-0033] Kjeldsen KU , Schreiber L , Thorup CA , Boesen T , Bjerg JT , Yang T , Dueholm MS , Larsen S , Risgaard‐Petersen N , Nierychlo M *et al*. 2019. On the evolution and physiology of cable bacteria. Proceedings of the National Academy of Sciences, USA 116: 19116–19125. 10.1073/pnas.1903514116 PMC6754541 31427514

[nph17415-bib-0034] Kumar U , Kumar Nayak A , Shahid M , Gupta VVSR , Panneerselvam P , Mohanty S , Kaviraj M , Kumar A , Chatterjee D , Lal B *et al*. 2018. Continuous application of inorganic and organic fertilizers over 47 years in paddy soil alters the bacterial community structure and its influence on rice production. Agriculture, Ecosystems & Environment 262: 65–75.

[nph17415-bib-0035] Lamers LP , Govers LL , Janssen IC , Geurts JJ , Van der Welle ME , Van Katwijk MM , Van der Heide T , Roelofs JG , Smolders AJ . 2013. Sulfide as a soil phytotoxin – a review. Frontiers in Plant Science 4: 268. 23885259 10.3389/fpls.2013.00268 PMC3717504

[nph17415-bib-0036] Li C , Reimers CE , Chapman JW . 2020. Microbiome analyses and presence of cable bacteria in the burrow sediment of *Upogebia pugettensis* . Marine Ecology Progress Series 648: 79–94.

[nph17415-bib-0037] Lin L , Liu W , Zhang M , Lin X , Zhang Y , Tian Y . 2019. Different height forms of *Spartina alterniflora* might select their own rhizospheric bacterial communities in southern coast of China. Microbial Ecology 77: 124–135. 29948019 10.1007/s00248-018-1208-y

[nph17415-bib-0038] Liu F , Wang Z , Wu B , Bjerg JT , Hu W , Guo X , Guo J , Nielsen LP , Qiu R , Xu M . 2021. Cable bacteria extend the impacts of elevated dissolved oxygen into anoxic sediments. The ISME Journal 15: 1551–1563. 33479492 10.1038/s41396-020-00869-8 PMC8114917

[nph17415-bib-0039] Liu H , Li J , Zhao Y , Xie K , Tang X , Wang S , Li Z , Liao Y , Xu J , Di H *et al*. 2018. Ammonia oxidizers and nitrite‐oxidizing bacteria respond differently to long‐term manure application in four paddy soils of south of China. Science of the Total Environment 633: 641–648. 10.1016/j.scitotenv.2018.03.108 29597161

[nph17415-bib-0040] Liu WJ , Zhu YG , Smith F , Smith S . 2004. Do phosphorus nutrition and iron plaque alter arsenate (As) uptake by rice seedlings in hydroponic culture? New Phytologist 162: 481–488.

[nph17415-bib-0041] Loy A , Lehner A , Lee N , Adamczyk J , Meier H , Ernst J , Schleifer K‐H , Wagner M . 2002. Oligonucleotide microarray for 16S rRNA gene‐based detection of all recognized lineages of sulfate‐reducing prokaryotes in the environment. Applied and Environmental Microbiology 68: 5064–5081. 12324358 10.1128/AEM.68.10.5064-5081.2002 PMC126405

[nph17415-bib-0042] Ma Q , Li J , Aamer M , Huang G . 2020. Increasing methane (CH_4_) emissions and altering rhizosphere microbial diversity in paddy soil by combining Chinese milk vetch and rice straw. PeerJ 8: e9653. 32832274 10.7717/peerj.9653 PMC7409806

[nph17415-bib-0043] Malkin SY , Meysman FJR . 2015. Rapid redox signal transmission by ‘Cable Bacteria’ beneath a photosynthetic biofilm. Applied and Environmental Microbiology 81: 948–956. 25416774 10.1128/AEM.02682-14 PMC4292484

[nph17415-bib-0044] Manz W , Amann R , Ludwig W , Wagner M , Schleifer K‐H . 1992. Phylogenetic oligodeoxynucleotide probes for the major subclasses of proteobacteria: problems and solutions. Systematic and Applied Microbiology 15: 593–600.

[nph17415-bib-0045] Martin BC , Alarcon MS , Gleeson D , Middleton JA , Fraser MW , Ryan MH , Holmer M , Kendrick GA , Kilminster K . 2019. Root microbiomes as indicators of seagrass health. FEMS Microbiology Ecology 96: fiz201. 10.1093/femsec/fiz201 31841144

[nph17415-bib-0046] Martin BC , Bougoure J , Ryan MH , Bennett WW , Colmer TD , Joyce NK , Olsen YS , Kendrick GA . 2018a. Oxygen loss from seagrass roots coincides with colonisation of sulphide‐oxidising cable bacteria and reduces sulphide stress. The ISME Journal 13: 707–719. 30353038 10.1038/s41396-018-0308-5 PMC6461758

[nph17415-bib-0047] Martin BC , Gleeson D , Statton J , Siebers AR , Grierson P , Ryan MH , Kendrick GA . 2018b. Low light availability alters root exudation and reduces putative beneficial microorganisms in seagrass roots. Frontiers in Microbiology 8: 2667. 29375529 10.3389/fmicb.2017.02667 PMC5768916

[nph17415-bib-0048] Martin BC , Middleton JA , Fraser MW , Marshall IPG , Scholz VV , Hausl B , Schmidt H . 2020. Cutting out the middle clam: lucinid endosymbiotic bacteria are also associated with seagrass roots worldwide. The ISME Journal 14: 2901–2905. 32929207 10.1038/s41396-020-00771-3 PMC7784995

[nph17415-bib-0049] Martin M . 2011. Cutadapt removes adapter sequences from high‐throughput sequencing reads. EMBnet.journal 17: 10–12.

[nph17415-bib-0050] Marzocchi U , Bonaglia S , van de Velde S , Hall PO , Schramm A , Risgaard‐Petersen N , Meysman FJ . 2018. Transient bottom water oxygenation creates a niche for cable bacteria in long‐term anoxic sediments of the Eastern Gotland Basin. Environmental Microbiology 20: 3031–3041. 29971901 10.1111/1462-2920.14349

[nph17415-bib-0051] McMurdie PJ , Holmes S . 2013. phyloseq: an R package for reproducible interactive analysis and graphics of microbiome census data. PLoS ONE 8: e61217. 23630581 10.1371/journal.pone.0061217 PMC3632530

[nph17415-bib-0052] Meharg AA . 2004. Arsenic in rice–understanding a new disaster for South‐East Asia. Trends in Plant Science 9: 415–417. 15337490 10.1016/j.tplants.2004.07.002

[nph17415-bib-0053] Mehraban P , Zadeh AA , Sadeghipour HR . 2008. Iron toxicity in rice (*Oryza sativa* L.), under different potassium nutrition. Asian Journal of Plant Sciences 7: 251–259.

[nph17415-bib-0054] Meysman FJR , Cornelissen R , Trashin S , Bonné R , Martinez SH , van der Veen J , Blom CJ , Karman C , Hou J‐L , Eachambadi RT *et al*. 2019. A highly conductive fibre network enables centimetre‐scale electron transport in multicellular cable bacteria. Nature Communications 10: 4120. 10.1038/s41467-019-12115-7 PMC6739318 31511526

[nph17415-bib-0055] Nguyen SG , Guevarra RB , Kim J , Ho CT , Trinh MV , Unno T . 2015. Impacts of initial fertilizers and irrigation systems on paddy methanogens and methane emission. Water, Air, & Soil Pollution 226: 309.

[nph17415-bib-0056] Nicolaisen MH , Risgaard‐Petersen N , Revsbech NP , Reichardt W , Ramsing NB . 2004. Nitrification–denitrification dynamics and community structure of ammonia oxidizing bacteria in a high yield irrigated Philippine rice field. FEMS Microbiology Ecology 49: 359–369. 19712286 10.1016/j.femsec.2004.04.015

[nph17415-bib-0057] Parks DH , Chuvochina M , Waite DW , Rinke C , Skarshewski A , Chaumeil P‐A , Hugenholtz P . 2018. A standardized bacterial taxonomy based on genome phylogeny substantially revises the tree of life. Nature Biotechnology 36: 996–1004. 10.1038/nbt.4229 30148503

[nph17415-bib-0058] Pérez‐Montaño F , Alías‐Villegas C , Bellogín RA , del Cerro P , Espuny MR , Jiménez‐Guerrero I , López‐Baena FJ , Ollero FJ , Cubo T . 2014. Plant growth promotion in cereal and leguminous agricultural important plants: From microorganism capacities to crop production. Microbiological Research 169: 325–336. 24144612 10.1016/j.micres.2013.09.011

[nph17415-bib-0059] Pernthaler J , Glöckner F‐O , Schönhuber W , Amann R . 2001. Fluorescence *in situ* hybridization (FISH) with rRNA‐targeted oligonucleotide probes. Methods in Microbiology 30: 207–226.

[nph17415-bib-0060] Pfeffer C , Larsen S , Song J , Dong M , Besenbacher F , Meyer RL , Kjeldsen KU , Schreiber L , Gorby YA , El‐Naggar MY *et al*. 2012. Filamentous bacteria transport electrons over centimetre distances. Nature 491: 218–221. 23103872 10.1038/nature11586

[nph17415-bib-0061] Pruesse E , Peplies J , Glöckner FO . 2012. SINA: accurate high‐throughput multiple sequence alignment of ribosomal RNA genes. Bioinformatics 28: 1823–1829. 22556368 10.1093/bioinformatics/bts252 PMC3389763

[nph17415-bib-0062] Putschew A , Scholz‐Böttcher BM , Rullkötter J . 1996. Early diagenesis of organic matter and related sulphur incorporation in surface sediments of meromictic Lake Cadagno in the Swiss Alps. Organic Geochemistry 25: 379–390.

[nph17415-bib-0063] Quast C , Pruesse E , Yilmaz P , Gerken J , Schweer T , Yarza P , Peplies J , Glöckner FO . 2013. The SILVA ribosomal RNA gene database project: improved data processing and web‐based tools. Nucleic Acids Research 41: D590–D596. 23193283 10.1093/nar/gks1219 PMC3531112

[nph17415-bib-0064] R Core Team . 2013. R: a language and environment for statistical computing, v. 3.6.0. Vienna, Austria: R Foundation for Statistical Computing. [WWW document] URL http://www.R‐project.org/.

[nph17415-bib-0065] Revsbech NP , Sorensen J , Blackburn TH , Lomholt JP . 1980. Distribution of oxygen in marine sediments measured with microelectrodes. Limnology and Oceanography 25: 403–411.

[nph17415-bib-0066] Risgaard‐Petersen N , Kristiansen M , Frederiksen RB , Dittmer AL , Bjerg JT , Trojan D , Schreiber L , Damgaard LR , Schramm A , Nielsen LP . 2015. Cable bacteria in freshwater sediments. Applied and Environmental Microbiology 81: 6003–6011. 26116678 10.1128/AEM.01064-15 PMC4551263

[nph17415-bib-0067] Risgaard‐Petersen N , Revil A , Meister P , Nielsen LP . 2012. Sulfur, iron‐, and calcium cycling associated with natural electric currents running through marine sediment. Geochimica et Cosmochimica Acta 92: 1–13.

[nph17415-bib-0068] Rothenberg SE , Anders M , Ajami NJ , Petrosino JF , Balogh E . 2016. Water management impacts rice methylmercury and the soil microbiome. Science of the Total Environment 572: 608–617. 10.1016/j.scitotenv.2016.07.017 PMC5099098 27450246

[nph17415-bib-0069] Rueden CT , Schindelin J , Hiner MC , DeZonia BE , Walter AE , Arena ET , Eliceiri KW . 2017. Image J2: ImageJ for the next generation of scientific image data. BMC Bioinformatics 18: 529. 29187165 10.1186/s12859-017-1934-z PMC5708080

[nph17415-bib-0070] Sandfeld T , Marzocchi U , Petro C , Schramm A , Risgaard‐Petersen N . 2020. Electrogenic sulfide oxidation mediated by cable bacteria stimulates sulfate reduction in freshwater sediments. The ISME Journal 14: 1233–1246. 32042102 10.1038/s41396-020-0607-5 PMC7174387

[nph17415-bib-0071] Schauer R , Risgaard‐Petersen N , Kjeldsen KU , Tataru Bjerg JJ , B Jørgensen Bo , Schramm A , Nielsen LP . 2014. Succession of cable bacteria and electric currents in marine sediment. The ISME Journal 8: 1314–1322. 24451206 10.1038/ismej.2013.239 PMC4030233

[nph17415-bib-0072] Schmidt H , Eickhorst T . 2014. Detection and quantification of native microbial populations on soil‐grown rice roots by catalyzed reporter deposition‐fluorescence in situ hybridization. FEMS Microbiology Ecology 87: 390–402. 24118011 10.1111/1574-6941.12232

[nph17415-bib-0073] Scholz VV , Meckenstock RU , Nielsen LP , Risgaard‐Petersen N . 2020. Cable bacteria reduce methane emissions from rice‐vegetated soils. Nature Communications 11: 1878. 10.1038/s41467-020-15812-w PMC7171082 32313021

[nph17415-bib-0074] Scholz VV , Müller H , Koren K , Nielsen LP , Meckenstock RU . 2019. The rhizosphere of aquatic plants is a habitat for cable bacteria. FEMS Microbiology Ecology 95: fiz062. 31054245 10.1093/femsec/fiz062 PMC6510695

[nph17415-bib-0075] Scilipoti S , Koren K , Risgaard‐Petersen N , Schramm A , Nielsen LP . 2021. Oxygen consumption of individual cable bacteria. Science Advances 7: eabe1870. 33568484 10.1126/sciadv.abe1870 PMC7875522

[nph17415-bib-0076] Seitaj D , Schauer R , Sulu‐Gambari F , Hidalgo‐Martinez S , Malkin SY , Burdorf LDW , Slomp CP , Meysman FJR . 2015. Cable bacteria generate a firewall against euxinia in seasonally hypoxic basins. Proceedings of the National Academy of Sciences, USA 112: 13278–13283. 10.1073/pnas.1510152112 PMC4629370 26446670

[nph17415-bib-0077] Shao J , He Y , Zhang H , Chen A , Lei M , Chen J , Peng L , Gu J‐D . 2016. Silica fertilization and nano‐MnO_2_ amendment on bacterial community composition in high arsenic paddy soils. Applied Microbiology and Biotechnology 100: 2429–2437. 26563550 10.1007/s00253-015-7131-y

[nph17415-bib-0078] Sobek S , Durisch‐Kaiser E , Zurbrügg R , Wongfun N , Wessels M , Pasche N , Wehrli B . 2009. Organic carbon burial efficiency in lake sediments controlled by oxygen exposure time and sediment source. Limnology and Oceanography 54: 2243–2254.

[nph17415-bib-0079] Sulu‐Gambari F , Seitaj D , Meysman FJ , Schauer R , Polerecky L , Slomp CP . 2016. Cable bacteria control iron–phosphorus dynamics in sediments of a coastal hypoxic basin. Environmental Science & Technology 50: 1227–1233. 26720721 10.1021/acs.est.5b04369

[nph17415-bib-0080] Tan YM , Dalby O , Kendrick GA , Statton J , Sinclair EA , Fraser MW , Macreadie PI , Gillies CL , Coleman RA , Waycott M *et al*. 2020. Seagrass restoration is possible: insights and lessons from Australia and New Zealand. Frontiers in Marine Science 7: 617.

[nph17415-bib-0081] Thorup C . 2019. Omics insights into cable bacteria metabolism. PhD thesis, Aarhus University, Aarhus, Denmark.

[nph17415-bib-0082] Trojan D , Schreiber L , Bjerg JT , Bøggild A , Yang T , Kjeldsen KU , Schramm A . 2016. A taxonomic framework for cable bacteria and proposal of the candidate genera Electrothrix and Electronema. Systematic and Applied Microbiology 39: 297–306. 27324572 10.1016/j.syapm.2016.05.006 PMC4958695

[nph17415-bib-0083] Ugarelli K , Laas P , Stingl U . 2019. The microbial communities of leaves and roots associated with turtle grass (*Thalassia testudinum*) and manatee grass (*Syringodium filliforme*) are distinct from seawater and sediment communities, but are similar between species and sampling sites. Microorganisms 7: 4. 10.3390/microorganisms7010004 PMC6352278 30587804

[nph17415-bib-0084] van de Velde S , Callebaut I , Gao Y , Meysman FJ . 2017. Impact of electrogenic sulfur oxidation on trace metal cycling in a coastal sediment. Chemical Geology 452: 9–23.

[nph17415-bib-0085] van de Velde S , Lesven L , Burdorf LD , Hidalgo‐Martinez S , Geelhoed JS , Van Rijswijk P , Gao Y , Meysman FJ . 2016. The impact of electrogenic sulfur oxidation on the biogeochemistry of coastal sediments: a field study. Geochimica et Cosmochimica Acta 194: 211–232.

[nph17415-bib-0086] Wang Q , Garrity GM , Tiedje JM , Cole JR . 2007. Naïve Bayesian classifier for rapid assignment of rRNA sequences into the new bacterial taxonomy. Applied and Environmental Microbiology 73: 5261–5267. 17586664 10.1128/AEM.00062-07 PMC1950982

[nph17415-bib-0087] Wickham H . 2016. ggplot2: Elegant graphics for data analysis. New York, NY, USA: Springer‐Verlag.

[nph17415-bib-0088] Wind T , Conrad R . 1997. Localization of sulfate reduction in planted and unplanted rice field soil. Biogeochemistry 37: 253–278.

[nph17415-bib-0089] Xiong Y , Guilbaud R , Peacock CL , Cox RP , Canfield DE , Krom MD , Poulton SW . 2019. Phosphorus cycling in Lake Cadagno, Switzerland: a low sulfate euxinic ocean analogue. Geochimica et Cosmochimica Acta 251: 116–135.

[nph17415-bib-0090] Zhu J , Peng H , Ji X , Li C , Li S . 2019. Effects of reduced inorganic fertilization and rice straw recovery on soil enzyme activities and bacterial community in double‐rice paddy soils. European Journal of Soil Biology 94: 103116.

[nph17415-bib-0091] Zinger L , Bonin A , Alsos IG , Bálint M , Bik H , Boyer F , Chariton AA , Creer S , Coissac E , Deagle BE *et al*. 2019. DNA metabarcoding – need for robust experimental designs to draw sound ecological conclusions. Molecular Ecology 28: 1857–1862. 31033079 10.1111/mec.15060

