# Supplementary material for: Cable bacteria at oxygen‐releasing roots of aquatic plants: a widespread and diverse plant–microbe association
Source: New Phytol. 2021 May 21;232(5):2138–51. doi: 10.1111/nph.17415 (PMC8596878; doi:10.1111/nph.17415)
Supplement: Supplementary file 1 — Fig. S1 Collection of Posidonia australis seeds and rhizobox design. Fig. S2 Photographs of sample locations without abundant plant‐associated cable bacteria. Fig. S3 Relative abundance of cable bacteria in rice fields (from 16S rRNA gene data). Fig. S4 Abundance of all bacteria, cable bacteria and Deltaproteobacteria along roots of P. australis seedlings. [file NPH-232-2138-s002.pdf]

New Phytologist Supporting Information

Article title: **Cable bacteria at oxygen-releasing roots of aquatic plants: a widespread and diverse plant-microbe association**

Authors: Vincent V. Scholz, Belinda C. Martin, Raïssa Meyer, Andreas Schramm, Matthew W. Fraser, Lars Peter Nielsen, Gary A. Kendrick, Nils Risgaard-Petersen, Laurine Burdorf and Ian P.G. Marshall

Article acceptance date: 9 April 2021

The following Supporting Information is available for this article:

Fig. S1 Collection of *Posidonia australis* seeds and rhizobox design.

Fig. S2 Photographs of sample locations without abundant plant-associated cable bacteria.

Fig. S3 Relative abundance of cable bacteria in rice fields (from 16S rRNA gene data).

Fig. S4 Abundance of all bacteria, cable bacteria and Deltaproteobacteria along roots of *P. australis* seedlings.

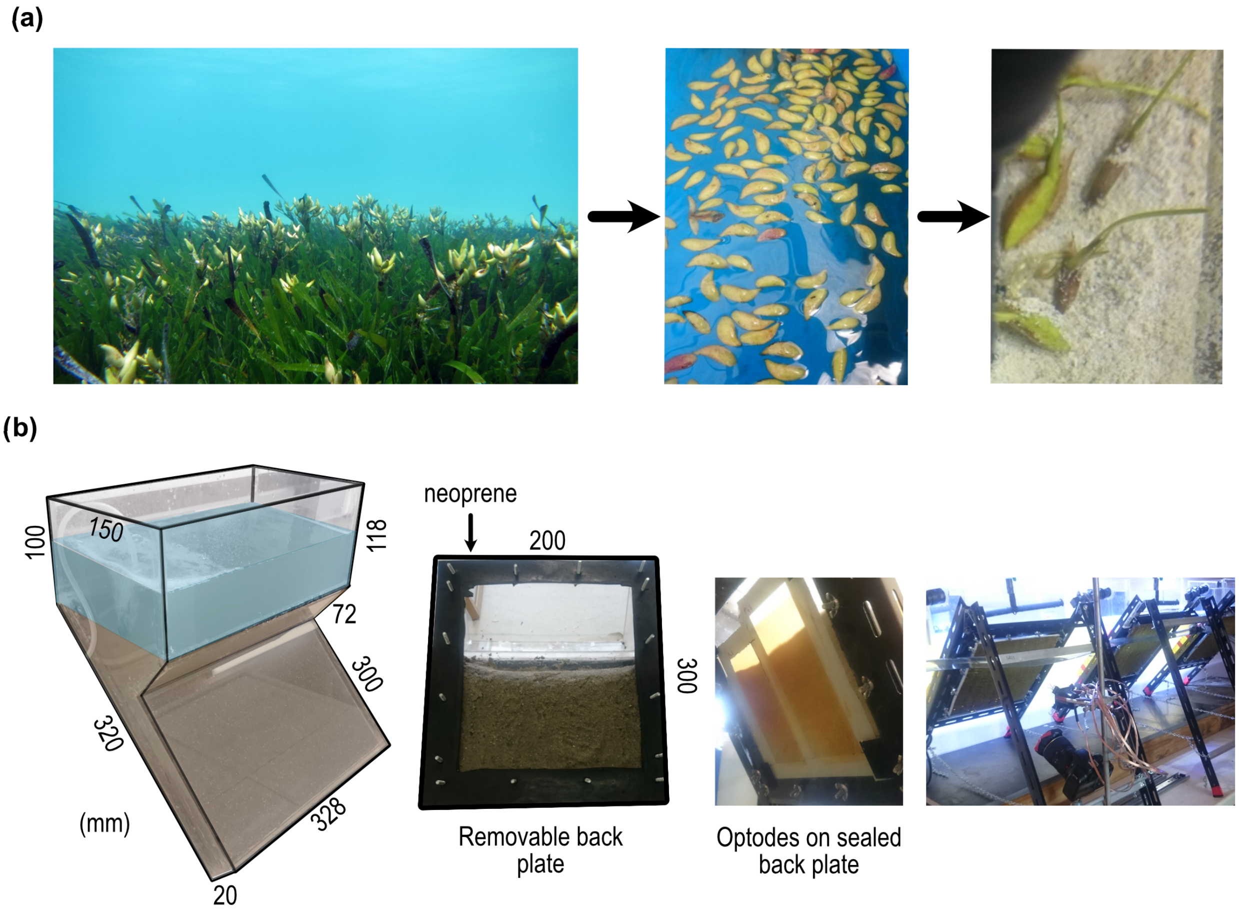

Fig. S1 Collection of *Posidonia australis* seeds and rhizobox design. (a) *P. australis* produces desiccation sensitive viviparous fruit once per year that directly develop into seedlings once detached from the parent plant. Because of this, fruits are collected directly from the plant and transferred to aquaria to naturally dehisce. Once fruits dehisce, the developing seedlings are negatively buoyant and fall directly to the bottom of the tank where they can be collected and then planted. (b) Design and dimensions of the rhizobox with a removable back plate to allow application and removal of optodes, as well as collection of plants without pulling the roots out of the sediment.

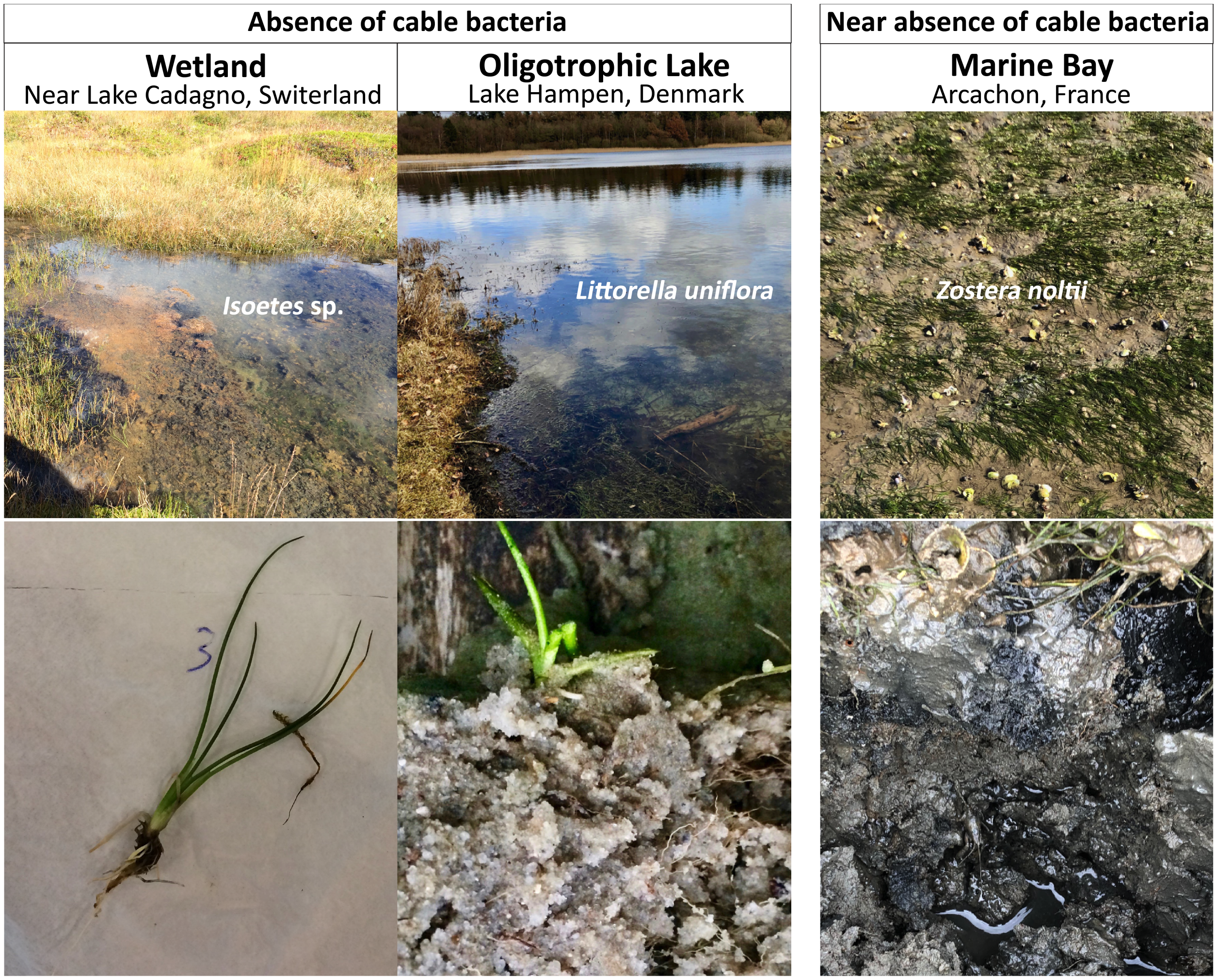

Fig. S2 Photographs of sample locations without abundant plant-associated cable bacteria. No cable bacteria were identified by fluorescence *in situ* hybridization and DNA sequencing around roots of *Isoetes* sp. at Lake Cadagno, Swizterland and *Littorella unilfora* in Lake Hampen, Denmark (left panel). Only one out of three roots samples from *Zostera noltii* in Arcachon showed the low abundance of an undefined *Ca.* Electrothrix species (12 reads) using 16S rRNA sequencing (right panel).

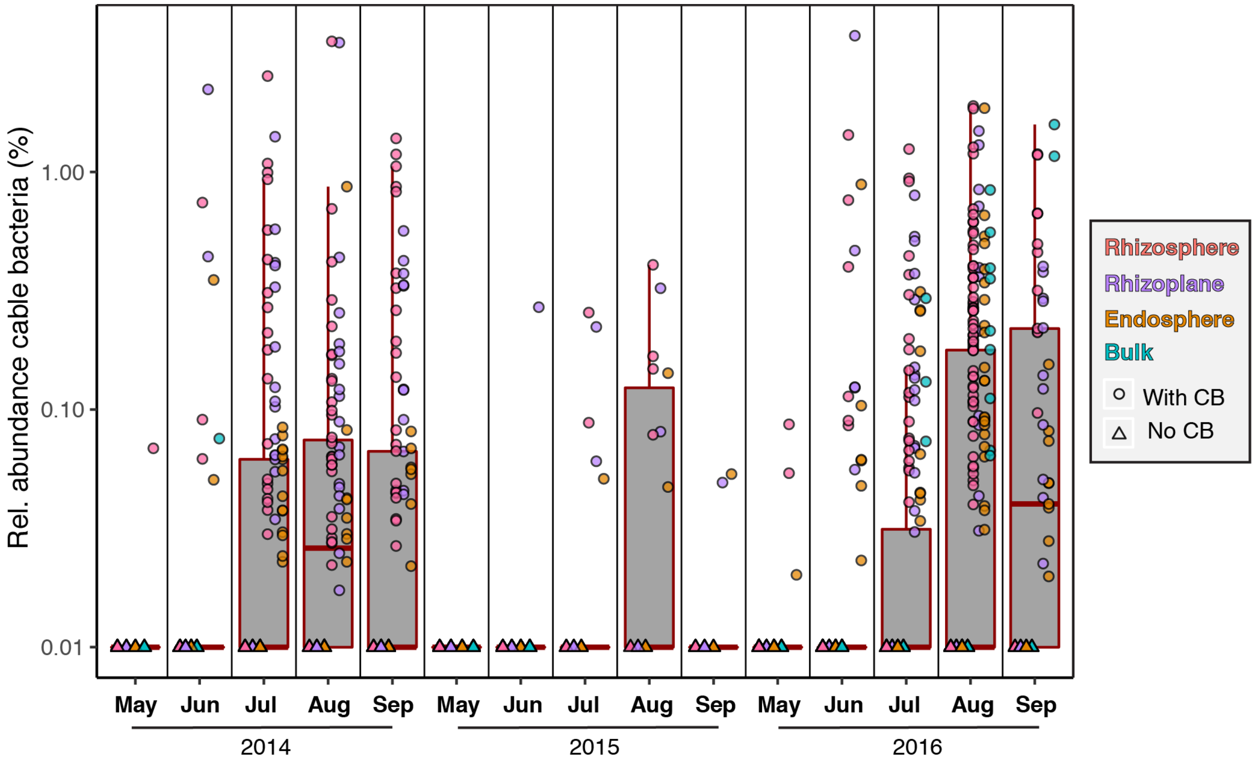

Fig. S3 Relative abundance of cable bacteria in rice fields (from 16S rRNA gene data). The relative cable bacteria abundance is plotted as a function of months during three consecutive growing seasons. For better presentation of the zero values, 0.01 was added to all samples. All sample points overlie the box-and-whisker plots. Grey boxes (the interquartile range, or IQR) indicate the 1st and 3rd quartiles, while the horizontal lines inside the boxes indicate the medians. The vertical lines extending from the tops of the grey boxes indicate the highest data points within the upper extent of the IQR plus 1.5 times the IQR. Note the absence of the whiskers extending from the lower quartiles due to the the high number of zero values in the dataset. Note also the logarithmic scale on the y-axis.

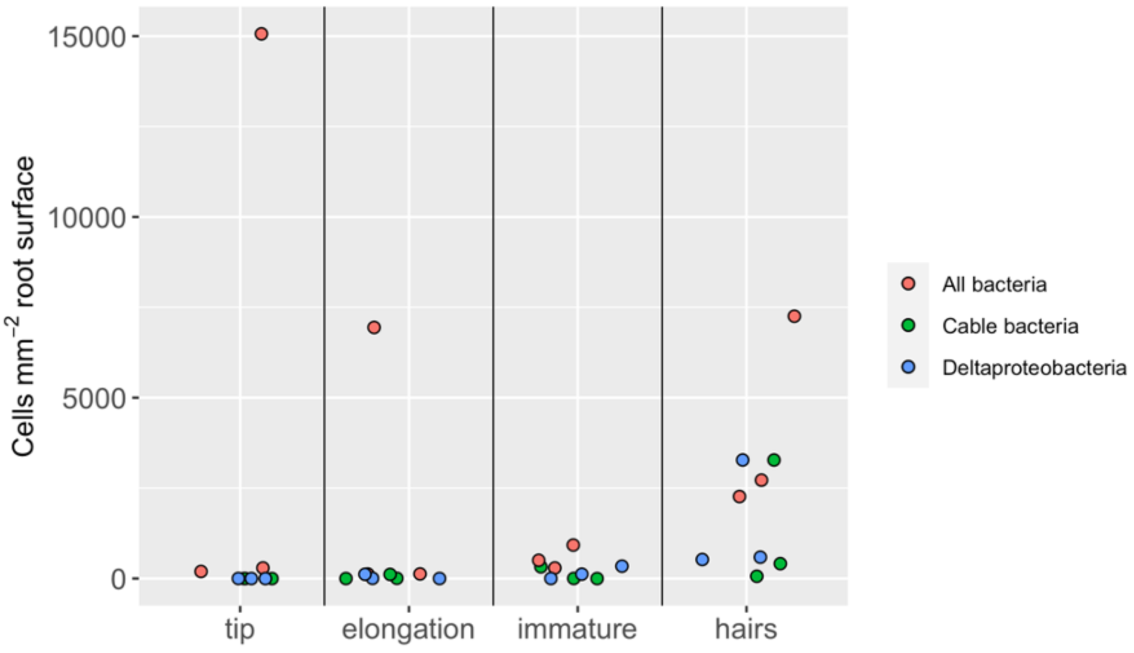

Fig S4 Abundance of all bacteria, cable bacteria and Deltaproteobacteria along roots of *P. australis* seedlings. Data were acquired by fluorescence *in situ* hybridization counts of cells hybridized with probes EUB-mix (all bacteria), DSB706 (cable bacteria) and Delta495a-c (Deltaproteobacteria). The datapoints of cable bacteria are the same as displayed in Fig. 6.
